# Multisensory Interplay Reveals Crossmodal Influences on ‘Sensory-Specific’ Brain Regions, Neural Responses, and Judgments

**DOI:** 10.1016/j.neuron.2007.12.013

**Published:** 2008-01-10

**Authors:** Jon Driver, Toemme Noesselt

**Affiliations:** 1 UCL Institute of Cognitive Neuroscience, University College London, 17 Queen Square, London WC1N 3AR, UK; 2 Department of Neurology II, Otto-von-Guericke-Universität, Leipziger Str. 44, 39120 Magdeburg, Germany

## Abstract

Although much traditional sensory research has studied each sensory modality in isolation, there has been a recent explosion of interest in causal interplay between different senses. Various techniques have now identified numerous multisensory convergence zones in the brain. Some convergence may arise surprisingly close to low-level sensory-specific cortex, and some direct connections may exist even between primary sensory cortices. A variety of multisensory phenomena have now been reported in which sensory-specific brain responses and perceptual judgments concerning one sense can be affected by relations with other senses. We survey recent progress in this multisensory field, foregrounding human studies against the background of invasive animal work and highlighting possible underlying mechanisms. These include rapid feedforward integration, possible thalamic influences, and/or feedback from multisensory regions to sensory-specific brain areas. Multisensory interplay is more prevalent than classic modular approaches assumed, and new methods are now available to determine the underlying circuits.

## Main Text

### Introduction

Sensory processing and perception have been studied intensively for decades, in both neuroscience and psychology. But most traditional research considered just a single sensory modality at a time (e.g., vision or audition or touch). By contrast, real-world situations often stimulate several of our senses concurrently. Moreover, subsets of the incoming stimulation across different modalities arise from common external objects or events, as when we both see and feel an object in our hand or both see and hear a person talking or a car moving.

In psychology, it has long been known that perceptual judgments can reflect combined information from multiple senses (Welch and Warren, 1986; Spence and Driver, 2004). Moreover, neuroscience has identified various “multisensory” brain regions as convergence zones, where neurons receive afferent inputs from several senses and combine these according to various constraints. But in recent years the field of multisensory research has expanded and altered radically with the realization that multisensory influences are much more pervasive than classical views assumed and may even affect brain regions, neural responses, and judgments traditionally considered modality specific. Here we consider such cases, in which multisensory effects can arise for apparently sensory-specific processes or perceptions. We will often refer to multisensory “interplay” rather than the commonly used “integration,” so as to include cases where one modality might affect another without necessarily always implying a single unified percept. We focus primarily on human perceptual studies but refer to important animal work as relevant background.

### Behavioral and Perceptual Consequences of Multisensory Interplay in Humans

Classic examples of multisensory perceptual “illusions” include spatial ventriloquism (mislocalization of sounds toward temporally correlated but displaced visual events), auditory driving (misperception of visual events as having the temporal frequency of apparently related auditory events), and the McGurk effect (perception of speech sounds influenced by seen lip movements). See Calvert et al. (2004), Macaluso and Driver (2005), Spence and Driver (2004), and Vroomen and de Gelder (2004) for more extensive reviews of such perceptual phenomena.

In such cases, information is typically provided by two or more different senses about one particular external property. The textbook multisensory effects are traditionally considered to be misleading illusions. But an increasingly influential view is that they reflect combined use of information from the separate modalities to yield a joint estimate of an external property. Thus, location information from two or more modalities may be jointly considered when estimating the location of an apparently multisensory event. Such joint estimates may be “optimal” in a formal sense, weighting each modality's contribution by its reliability/variability for the property concerned (e.g., Alais and Burr, 2004; Ernst and Bulthoff, 2004; Helbig and Ernst, 2007).

When more than one input is provided within each of several senses, as will often apply in the real world, a further issue arises concerning *which* particular inputs from one sense should be jointly weighted together with which particular selection of inputs from other senses. Spatial, temporal, and semantic/associative relations may be critical in constraining such selective combination of related or jointly parsed subsets from multiple inputs to multiple senses. For recent examples regarding spatial constraints, see Frassinetti et al. (2002), for temporal constraints, see Fendrich and Corballis (2001) and Recanzone (2003), for combined spatiotemporal constraints, see Zampini et al. (2003); see also Vatakis and Spence (2007) for further discussion.

By contrast, other recent examples of multisensory influences on perception arguably demonstrate a different type of phenomenon. Rather than several modalities providing independent samples about the *same* external property, stimulation in one modality may now affect judgments of a property that logically applies only to another modality. Thus, presence/absence judgments concerning only one modality (e.g., for vision) can be enhanced when a sound co-occurs at the location of the visual event to be detected, affecting visual sensitivity rather than merely criterion (e.g., McDonald et al., 2000). Touch at a given location can even improve judgments of visual color nearby, although touch itself cannot convey color (see Spence et al., 2004). Here, events in a modulating modality may render a particular region of space (and/or time) salient for another modality, to facilitate modality-specific processing for that time or place in the latter modality (Driver and Spence, 2000; Frassinetti et al., 2002; Lovelace et al., 2003; McDonald et al., 2000; Vroomen and de Gelder, 2000). Such results provide an initial behavioral hint that events in one modality may sometimes affect sensory-specific processing for another modality, as now also indicated by some of the neural measures we consider later.

Other recent examples of behavioral multisensory effects extend the classic phenomenon of auditory driving (Shipley, 1964) with the auditory-flash illusion. Shams et al. (2000) reported that a single flash can be misperceived as two flashes if paired with two beeps (see also Arden et al., 2003; Mishra et al., 2007; Shams et al., 2001; Watkins et al., 2006). Berger et al. (2003) showed that when multiple sounds produce the impression of more visual events than actually occurred, visual orientation discriminations can improve objectively (even though the sounds do not provide any orientation information), analogously to when more visual events actually did occur. Thus, multisensory interplay can affect sensory-specific judgments.

### Neural Studies of Multisensory Interactions: Traditional Focus on Multisensory Convergence Zones and Recent Findings on This Topic

**Figure 1:**
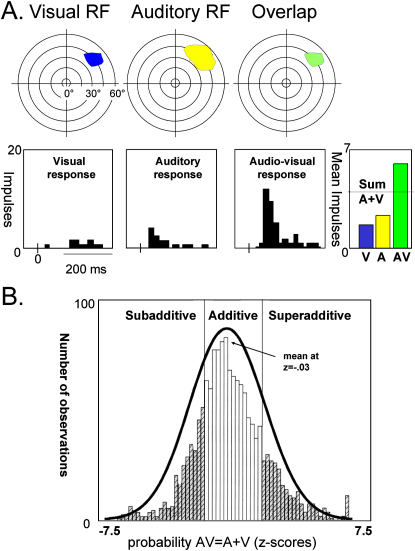
Response Properties of Multisensory Neurons (A) Response properties of a putatively illustrative multisensory neuron, in deep superior colliculus, which in this case shows the often-discussed nonlinearly superadditive pattern of firing. That is, the response for combined visual and auditory stimulation, with a particular spatiotemporal relation, greatly exceeds the sum of the responses to each modality alone (adapted from Stein et al., 2004, by permission of Oxford University Press). (B) Distribution of z scores for a population of sampled neurons within deep layers of the cat superior colliculus, where z scores relate to firing rates for combined audiovisual stimulation, as compared with summed unisensory auditory and unisensory visual responses (©2007 by Oxford University Press, reprinted with permission). Note that while the z scores are distributed, with some neurons showing nonlinear multisensory responses (shaded columns), as often emphasized in the literature and as exemplified in (A), the distribution does in fact appear normal around zero, indicating that the average (and majority) population response of SC neurons may be additive/linear, even though some individual neurons depart from this (adapted from Stein et al., 2004). See later text for possible implications for fMRI research, where nonlinear criteria have often been proposed for assessing multisensory population responses, but may be overly restrictive.

**Figure 2:**
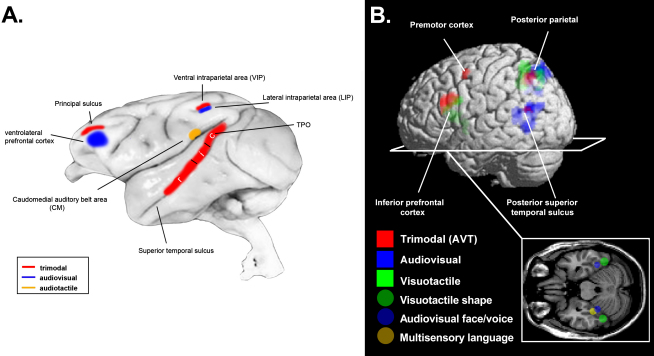
Anatomy of Cortical Multisensory Areas (A) Schematic overview of the anatomy of some cortical multisensory areas derived from anatomical, electrophysiological, and functional imaging data in nonhuman primates. (B) Illustration of candidate human multisensory cortical regions (found within prefrontal, parietal, and premotor cortex, plus superior temporal sulcus), derived from the overlap of BOLD responses to passive unisensory stimulation with brief auditory, visual, or tactile events in 12 healthy adult subjects, shown as a surface rendering for the left hemisphere. This reflects a new analysis of data from one experiment in our own fMRI work, but similar areas are implicated in many other human fMRI studies (e.g., see Macaluso and Driver, 2005). Cortical regions where visual and auditory responses overlap are shown in blue; those where visual and tactile responses overlap are shown in green; and regions showing a response to passive stimulation in any of these three modalities are shown in red. Similar regions also activated in right hemisphere. Because these depicted activations reflect BOLD responses induced merely by “simple” stimulation with brief events in one or another modality, we also depict (more schematically) additional cortical areas reported for combined multisensory stimuli, including face-voice combinations (Kriegstein et al., 2005), multisensory speech perception (Buchel et al., 1998), plus an area involved in visual-tactile shape interactions in lateral-occipital complex (LOC; Amedi et al., 2002).

Converging evidence from single-cell studies, tracing work, and recent human neuroimaging indicate numerous multisensory convergence zones in the brain (e.g., Mesulam, 1998; Kaas and Collins, 2004; Wallace et al., 2004); that is, brain regions where neurons receive afferent inputs from multiple senses. This has now been observed for numerous cortical and subcortical regions (see Figures 1 and 2 for examples).

Subcortically, deep layers of the superior colliculus (SC)—in addition to other subcortical regions such as basal ganglia (e.g., Nagy et al., 2006)—receive inputs from somatosensory, auditory, and visual areas (e.g., Meredith and Stein, 1983, 1986; Stein, 1978; Stein and Arigbede, 1972). Numerous influential studies by Stein and colleagues investigated multisensory interplay in cat SC neurons. When stimulating more than one sense, activity in deep SC neurons can depend on the spatial and temporal relation between inputs to different senses. Super- or subadditive responses can sometimes be observed for multisensory costimulation, as compared to stimulating either sense individually (Figure 1A), though this is far from ubiquitous (Figure 1B). Pioneering studies reported that multisensory interplay at the cellular level can be largest when each unisensory input alone elicits a relatively weak neural discharge, as for less intense stimuli (“inverse effectiveness,” e.g., Stein and Meredith, 1993). Others argue that this may reflect ceiling effects when using stronger unisensory inputs, or constraints from the dynamic range of neural firing (e.g., Schnupp et al., 1998; Holmes, 2007). It has also been suggested that structures such as the SC might be more involved in motoric spatial orienting than in perception per se (e.g., Redgrave et al., 1996).

Early studies often used anesthetized animals, though subsequent work used awake animals and sought to relate cellular findings to multisensory effects on orienting behavior (Stein et al., 1988; Stein and Meredith, 1990). Multisensory effects within SC neurons can have a relatively late onset and may depend on influences from cortical areas (Jiang et al., 2001), because reversible lesioning of multisensory cortex (ectosylvian and rostral lateral suprasylvian sulcus, rLS) in cats can eliminate multisensory effects in adult SC. Removal of multisensory ectosylvian cortex and rLS during early developmental in cats disrupts development of multisensory SC properties (Jiang et al., 2007; Wallace et al., 2006).

Turning to cortical regions in primates (see Kaas and Collins, 2004, for review), the upper bank of the superior temporal sulcus (TPO, see Figures 2A and 2B) is known to have bidirectional connections with unisensory auditory, visual, and somatosensory cortices (e.g., see Cusick, 1997; Padberg et al., 2003; Schmahmann and Pandya, 1991) and to contain multisensory neurons (e.g., Bruce et al., 1981; Barraclough et al., 2005; see also Beauchamp, 2005a).

Several regions within parietal cortex (e.g., areas VIP/LIP; see Figure 2A) are also known to receive input from sensory-specific cortices for different modalities. They may be involved in representing multisensory space relative to various body parts, in distinct spatial reference frames (see e.g., Cohen and Andersen, 2004; Duhamel et al., 1998; Maravita et al., 2003; Molholm et al., 2006; Sereno and Huang, 2006). Finally, specific premotor and prefrontal cortical regions have also been implicated in multisensory processing (see Figure 2A), with different subregions having specific interconnections with sensory-specific cortices (e.g., Barbas et al., 2005; Sugihara et al., 2006). Some direct connections have even been reported recently between prefrontal cortex and *primary* sensory cortices (Budinger et al., 2007; Wang and Burkhalter, 2007).

It should be noted that somewhat different criteria have been used to define a region as a multisensory convergence zone for different approaches. Anatomical tracing studies typically test for traceable connections with sensory-specific areas for more than one modality. Physiological single-cell studies consider the presence of responses to more than one modality when each is stimulated separately and/or responses during multisensory stimulation, compared to unisensory baselines, or when varying the temporal, spatial, or associative relation between costimulation in different modalities (see below). Finally, neuroimaging studies inherently assess only the more “macro” level of large-scale neural populations, with measures such as BOLD signal. Neuroimagers may therefore need to consider the possibility that a brain region seemingly responding to multiple modalities might comprise distinct interdigitated neural populations, each responding to only one of the various senses (see below). Convergence between multiple different approaches and measures is desirable and is now increasingly evident for the multisensory field.

### Multisensory Influences on ‘Sensory-Specific’ or Even Primary Cortical Areas

As noted in our introduction, there has been something of a revolution in multisensory research recently, due to the increasing realization that interplay between different senses can affect not only established multisensory convergence zones (see previous section) but may also affect brain regions, neural responses, and perceptual judgments traditionally considered to be sensory specific (i.e., concerning only vision or only audition or only touch, etc.). Such effects, on apparently unisensory levels of processing, contrast with the traditional view of sensory-specific areas feeding forward into higher multisensory convergence-zones (see previous section), with multisensory interplay traditionally thought to arise only for the latter.

The “new look” in this field now suggests that even classic sensory-specific areas (perhaps even primary cortices) can be influenced by multisensory interplay. There is some old, often overlooked evidence for this (Fishman and Michael, 1973; Morrell, 1972; Spinelli et al., 1968), but initial reports of apparent auditory responses for neurons in early visual cortex might have reflected nonspecific or confounding factors (e.g., arousal, pupil dilation, microsaccades caused by a sudden sound), due to technical limits at the time. But more recent studies using state-of-the-art methods now also indicate that some traditional sensory-specific brain regions, or early ERP modulations (sometimes within ∼30 ms of stimulus onset), can be influenced by multisensory interplay (see Ghazanfar et al., 2005; Hunt et al., 2006; Kayser et al., 2007; Lakatos et al., 2007; Molholm et al., 2002; Senkowski et al., 2005). The increasing flood of studies now indicating this has led to it rapidly emerging as the new consensus. On the other hand, each particular case needs to be judged on its own merit, and various potential confounds and interpretative issues must often be dealt with.

For instance, when dealing with fMRI cases, it should be noted that fMRI studies in other domains outside the multisensory field (e.g., concerning imagery or anticipatory attention) show that BOLD signals in sensory regions can be influenced by factors such as attention or imagery, even without any external stimulus (e.g., Kastner et al., 1999; Slotnick et al., 2005). Hence some of the oft-cited fMRI examples of apparent multisensory influences on unisensory cortex (e.g., Calvert et al., 1997) might conceivably reflect imagery, such as imagining corresponding speech sounds when seeing a very small set of lip movements that silently mouth spoken digits (cf. Goyal et al., 2006). Interpretation of some of the early neuroimaging findings remains unclear in such respects. Other examples are less susceptible to imagery accounts, yet might involve attentional influences instead (e.g., Macaluso et al., 2000). Indeed, a whole new field of research has opened up concerning multisensory links in selective attention (e.g., see Spence and Driver, 2004; Macaluso and Driver, 2005).

Several fMRI studies have now reported modulation of traditional “unisensory” cortical areas (usually defined as occipital-visual, postcentral-tactile, or temporal-auditory) due to multisensory costimulation (e.g., Amedi et al., 2002; Buchel et al., 1998; Calvert et al., 1999, 2001; Kriegstein et al., 2005; Macaluso et al., 2000; Martuzzi et al., 2007; Miller and D'Esposito, 2005; Watkins et al., 2006). Miller and D'Esposito (2005) reported modulation of auditory cortex when subjects perceived audiovisual speech stimuli as (a)synchronous. Localization to specific areas within “primary” auditory cortex proper (which comprises several subregions) can sometimes be questioned for normalized group studies at relatively low fMRI resolution in humans. Using higher-resolution fMRI in monkeys, together with separate mapping of specific auditory-cortex regions, Kayser et al. (2005) observed increased BOLD signal in secondary auditory cortex due to tactile costimulation. Even primary auditory areas were affected during visual costimulation (Kayser et al., 2007). Although these two studies had impressive anatomical resolution, it may be important to combine this with paradigms drawn from the psychological multisensory literature in the future, as most of the initial monkey fMRI studies did not as yet manipulate the relation (e.g., temporal, spatial, or associative) between multisensory stimulation, rather just the presence/absence (or salience) of costimulation in a second modality; nor did they measure perception. Moreover, the apparently different pattern of results for audiovisual costimulation versus audiotactile costimulation in auditory cortex hints at potentially different circuits for different pairings of modalities (see below). Nevertheless, this work provides powerful fMRI techniques for future animal studies.

Several different analysis strategies have been used in multisensory fMRI studies to date, for both humans and monkeys. Some studies (e.g., Calvert et al., 2001; Kayser et al., 2007) were influenced by the pioneering cellular SC studies of Stein and colleagues (see earlier section and Figure 1A), and so tested for BOLD signals in response to multisensory costimulation that were superadditive (or subadditive) with respect to the sum of both unisensory baselines. But some more recent cellular studies indicate that linear responses may actually be quite common in multisensory neurons receiving converging inputs from different senses, with strict super- or subadditivity being observed more rarely at the population level (Stein et al., 2004; see Figure 1B). Accordingly, many fMRI researchers have now adopted different analysis criteria. These include the “max criterion,” which identifies multisensory influences when the BOLD signal for costimulation of two modalities exceeds the larger of two unisensory baselines; or else a “mean criterion,” on which the multisensory response just has to exceed the mean of both unisensory responses (Beauchamp, 2005b). A further consideration for fMRI studies again concerns the relatively limited spatial resolution of current methods. Recent invasive physiological studies indicate that neurons receiving afferents from multiple senses might be found at the border of some strictly unisensory visual and auditory areas (e.g., Wallace et al., 2004; see also Beauchamp et al., 2004, for a high-resolution fMRI study). Large fMRI voxels might potentially intermix such different populations (Laurienti et al., 2005). This potential limitation of fMRI is by no means unique to the multisensory topic, and several fMRI approaches originally introduced for other topics might be used to circumvent it. For instance, it is often argued that “priming” effects, or BOLD repetition-suppression measures, can be used to assess whether a given region contains different interdigitated neural populations or, instead, a single population that generalizes across a particular property (e.g., Grill-Spector et al., 2006). Such generalization might (or might not) be found to apply across modalities, if tested this way for a given candidate multisensory area. Moreover, by manipulating the specific temporal, spatial, or semantic/associative relation between sensory inputs to different senses, one can test with fMRI whether a given brain region is sensitive or not to that particular crossmodal relation. In this way, more specific predictions can be tested than just whether a given region responds to two or more modalities overall at the resolution tested or whether costimulation differs from unisensory stimulation.

Turning to ERP or MEG studies, which can provide fine-grained temporal resolution (but less spatial information), most studies reported so far used simple present/absent (co)stimulation paradigms, manipulating whether a second modality was costimulated along with a first and assessing the impact on ERPs in response to a primary event type in the first modality (Giard and Peronnet, 1999; Molholm et al., 2002, 2004 ; Murray et al., 2005; Teder-Salejarvi et al., 2002, 2005). ERPs due to combined audiovisual stimulation might thus be compared to summed unisensory ERPs to test for any nonlinearities. Depending on the exact paradigm, preparatory states can introduce a potential artifact into such ERP comparisons (Teder-Salejarvi et al., 2002). Relatively few studies have avoided this (Busse et al., 2005; Talsma and Woldorff, 2005) and typically reported somewhat later modulations due to multisensory costimulation (e.g., arising at ∼180 ms rather than at ∼30 ms poststimulus onset). Moreover, the earliest (∼30 ms) ERP effects of combined multisensory stimulation do not appear sensitive to the relative location of stimuli in the different senses (Murray et al., 2005), unlike the spatial multisensory phenomena observed at the cellular (Stein and Meredith, 1993), fMRI (e.g., Macaluso et al., 2000; Macaluso and Driver, 2005), and behavioral levels (Frassinetti et al., 2002; Spence and Driver, 2004). An influence that simply reflects the presence/absence of costimulation in a second modality, rather than the particular relation between stimuli in different senses, might arguably reflect some nonspecific influence such as rapid alerting or arousal. Such effects might nevertheless still reflect a genuine influence between the senses (e.g., some form of rapid anatomical projection from one modality to another, see below).

Other multisensory ERP studies have manipulated additional factors beyond mere presence/absence of additional costimulation in a second modality. Kennett et al. (2001) found that the visual N1 component (and possibly the P1) was enhanced when tactile stimulation occurred at the same rather than different location to a visual event. McDonald et al. (2003) found that the visual P1 component could be modified by the relative location of a task-irrelevant sound with respect to the visual event. More recently, McDonald et al. (2005) reported amplitude modulation of the visual P1, for visual stimuli whose temporal properties were illusorily shifted due to sounds. Finally, using visual-tactile stimuli, a latency shift of the visual P1 component was reported for attend-visual relative to attend-tactile conditions (Vibell et al., 2007).

Unlike those studies (such as Kennett et al., 2001; McDonald et al., 2003) that presented a “cue” event in one modality prior to a “target” event in another sense, Busse et al. (2005) studied effects of task-irrelevant auditory *co*stimulation on a visual spatial attention task. Comparing attended versus unattended visual stimuli when combined with synchronous sounds (relative to unisensory attended versus unattended visual stimuli) revealed modulation over frontal electrodes, interpreted as indicating that visual attention may “spread” to the irrelevant auditory modality for temporally related stimuli. Talsma and Woldorff (2005) also used an audiovisual spatial attention task and reported a positivity starting ∼100 ms after stimulus onset, for audiovisual stimuli relative to the sum of unisensory stimuli. Talsma et al. (2007) reported modulation of the auditory P50 arising only when subjects attended to both audition and vision, highlighting a possible interdependence between multisensory interplay and attention (see also Talsma et al., 2006).

**Figure 3:**
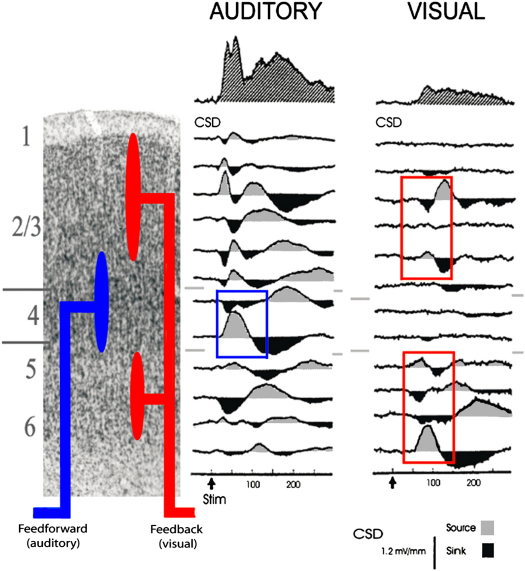
Electrophysiological Effects of Visual and Auditory Stimulation in Macaque Auditory Cortex Illustration of the laminar current-source densities (CSDs), found in a subregion of auditory association cortex posterior-lateral to A1 in monkeys, due to visual and auditory stimulation when recorded with multicontact electrodes (intercontact distance 150 μm) (reprinted from Schroeder and Foxe, 2002, by permission of Elsevier). CSDs reflect local postsynaptic potential (PSP) patterns. In the CSD profile, downward deflections (dark shaded) signify net extracellular current sinks (representing inward transmembrane currents) while upward deflections (gray shaded) indicate net extracellular current sources (representing outward currents). Sinks and sources are associated with local de- or hyperpolarization in local neuronal ensembles, respectively. Blue boxes emphasize CSD configurations due to auditory stimuli that reflect the initial excitatory response at layer 4. Red boxes reflect CSD configurations due to visual stimuli above and below layer 4 (see also illustrative diagram of feedforward/feedback connections in leftmost column overlaid on the six layers of auditory association cortex). These results strongly suggest that both auditory and visual stimuli are processed in this particular “auditory” area. However, the underlying neural mechanisms are different and indicative of feedforward versus feedback processing, respectively.

Moving beyond the relatively “macro” measures of fMRI or EEG in humans, more invasive recordings in animals have revealed strong evidence for multisensory responses in brain regions that would traditionally have been considered modality specific. Schroeder and colleagues (Ghazanfar and Schroeder, 2006; Hackett et al., 2007; Schroeder and Foxe, 2002; Schroeder et al., 2003; Smiley et al., 2007) reported multisensory convergence for some areas in and around “auditory” cortex, as described briefly below. They studied the laminar profile and timing of these influences, which can provide a particularly direct way to distinguish feedforward, lateral, and feedback routes (see Figure 3). Other reports indicate that posture (e.g., eye-in-orbit) may modulate responses to auditory signals in A1 (Fu et al., 2004; Werner-Reiss et al., 2003). More recently, Lakatos and colleagues reported that tactile stimuli can modulate the initial response to auditory stimuli in macaque primary auditory cortex (A1; Lakatos et al., 2007). Given that tactile input here enters layers I–III (see also Cappe and Barone, 2005; Budinger et al., 2006, 2007), this might be considered a “modulatory” rather than driving influence, in the terms of Felleman and van Essen (1991) and Rouiller et al. (1991). Taken together, numerous recent physiological studies indicate nonauditory influences on low-level and even primary auditory cortex in animals (e.g., Bizley et al., 2007; Brosch et al., 2005; Fu et al., 2004; Ghazanfar et al., 2005; Kayser et al., 2007; Lakatos et al., 2007; Werner-Reiss et al., 2003; see Kayser and Logothetis, 2007, for review).

### Different Accounts and Possible Architectures for Multisensory Influences on ‘Sensory-Specific’ Processing

The “new look” emerging in the multisensory field, with increasing reports of multisensory influences upon brain areas, neural responses, and even perceptual judgments traditionally considered sensory specific (see above), has led to several new explanatory proposals (see Figure 5 for schematic examples of these accounts). These are often considered as “rival” views in the current literature, but in fact may not be mutually exclusive. Some of the proposals may be correct for one class of phenomena, alternative proposals for a different set of findings, and so on. The various architectures proposed may coexist.

#### Account A (for ‘All Multisensory’)

Ghazanfar and Schroeder (2006) recently posed the provocative question “Is necortex essentially multisensory?” On an extreme version of this view (which they may not be advocating), all brain areas would be equal, in the sense that all are multisensory (or at least all contain some multisensory interneurons, see below). But many studies show regional preferences for one modality more than others (Macaluso and Driver, 2005; Van Essen et al., 1992), or for certain pairings of two modalities more than pairings of another two (Ghazanfar and Schroeder, 2006). Functional specialization is a very basic principle of brain organization (e.g., Krubitzer et al., 1997), so it seems implausible that all areas are multisensory in an undifferentiated sense.

Nevertheless, as noted toward the end of our previous section, recent invasive recordings confirm sensory responses to more than one modality within several regions closely adjacent to sensory-specific cortex, particularly in and around auditory cortex (Ghazanfar et al., 2005; Ghazanfar and Schroeder, 2006; Lakatos et al., 2007; Schroeder and Foxe, 2002; Wallace et al., 2004). One interpretation would be that even “primary” cortex can be intrinsically multisensory (at least for audition, e.g., Brosch et al., 2005). On the other hand, auditory cortex comprises numerous subregions, and some of these may remain specifically auditory or may instead be bimodal for regions bordering either with visually responsive regions (as for parts of the STS) or with tactile areas (as near SII).

Further neuroanatomical evidence demonstrates connections that might enable multisensory interplay to arise even at subcortical thalamic levels, as in gerbils (Budinger et al., 2006). In macaques, multisensory thalamic input may vary with the hierarchical level of the cortical area involved (Hackett et al., 2007; Hashikawa et al., 1991), with auditory areas such as CM receiving larger input from multisensory nuclei than A1. Recent invasive recordings suggest that tactile stimulation can modulate the first neural response in A1 via a phase-dependent modulatory influence in superficial cortical layers (Lakatos et al., 2007). It has been hypothesized that calbindin-positive neurons in the thalamus may provide a possible source for such modulation (see also Hackett et al., 2007). Further research is required to test this and for any generalization to other modality pairings. More generally, while possible roles for the thalamus in multisensory interplay are emerging in the animal literature, these have received less attention to date in the human literature, in part because of the more macro neural measures typically used in humans (though see Martínez et al., 1999; Noesselt et al., 2002).

Possible cortico-cortical routes for direct influences between senses (see Figure 5Aii) arise from the recently described monosynaptic connections between primary auditory cortex into primary visual cortex (macaque: Clavagnier et al., 2004; Falchier et al., 2002; ferret: Bizley et al., 2007) or between primary auditory cortex and somatosensory and olfactory cortex (gerbil: Budinger et al., 2006). Another study reported bidirectional fibers between auditory belt areas and primary visual cortex in macaques (Rockland and Ojima, 2003). Such connections may directly link sensory-specific cortices without involvement of intervening multisensory regions (see also Cappe and Barone, 2005). On the other hand, such connections seem relatively sparse, especially between primary areas. Current data from macaques suggest fewer direct connections between sensory-specific cortices than for feedback connections (see later section) to those from conventional multisensory areas, such as STS (Falchier et al., 2002). Moreover, the function(s) of the direct connections between primary cortices established to date still remain unclear. These might involve relatively nonspecific modulations (e.g., arousal, alerting, or overall weighting of one modality relative to another) rather than effects that depend on particular relations (e.g., relative location or semantic/associative links) between stimuli in different modalities.

ERP reports (e.g., Giard and Peronnet, 1999; Molholm et al., 2002; Senkowski et al., 2007) of relatively early influences (within ∼30 ms from stimulus onset) due to costimulation in a second modality might conceivably reflect such direct connections and/or possible thalamic gating (de la Mothe et al., 2006). On the other hand, source localization remains imperfect with EEG (which can make it unclear whether primary cortex in particular is influenced), and as mentioned earlier some of the comparisons used to date within EEG studies have been critiqued (e.g., Teder-Salejarvi et al., 2002).

Despite the growing evidence for some direct inputs from another sense into classical “unisensory” areas, most results for such areas still demonstrate a strong preference for one particular modality over others. Terms such as “sensory-specific” might thus be retained for such areas, for which one particular sense is clearly predominant, albeit with new caveats given the recent findings. As we described above (and extend further below), some degree of input from other modalities may modulate responses to the predominant modality in such regions, either through “modulatory” response amplification in superficial layers (as in A1 for audiotactile stimulation) or directly through a driving input into granular layers (as in area CM); see Schroeder and Foxe (2002).

#### Account B (for ‘New Bimodal Brain Areas’)

Another possible way to incorporate the emerging consensus that multisensory effects can influence traditional “sensory-specific” regions would simply posit that newly identified multisensory convergence zones exist, earlier than previously thought. This can be considered a less extreme version of Account A. There may be transitional multisensory zones adjacent to sensory-specific cortex (see Beauchamp et al., 2004; Wallace et al., 2004). While this might provide a new parcellation, in some respects it may continue the traditional divide between unisensory and multisensory regions, simply adding more of the latter at earlier processing stages than classically considered. Some of the multisensory convergence zones identified by Schroeder and colleagues, in and around auditory cortex, show multisensory effects at rapid latencies that evidently reflect input into feedforward layers (Schroeder and Foxe, 2005). As a consequence, multisensory cortical effects could in principle arise (at least for such regions) much earlier in time than envisaged by traditional, strictly sequential models on which sensory-specific processing is completed first, followed only later by multisensory interplay. This might accord with the emphasis on early modulations in much of the recent EEG literature (notwithstanding the caveats and potential methodological critiques considered for EEG work above). Rapid multisensory interplay in such specific “bimodal” areas may be constrained by the different signal-transduction times that typify auditory, tactile, and visual stimuli (which virtually prohibit visual signals from modifying the initial cortical response to auditory or tactile stimuli synched with visual events in the outside world). Such considerations might potentially explain why Kayser et al. (2005, 2007) observed different BOLD-modulation patterns in macaque auditory cortex for audio-tactile costimulation, as compared with audio-visual stimuli. Moreover, distinct auditory areas may receive input from different visual areas (Bizley et al., 2007), suggesting that specific bimodal areas might be further characterized by the functional “closeness” of subprocesses within different modalities (Bizley et al., 2007; Hackett et al., 2007; Smiley et al., 2007).

More generally, the proliferation of bimodal areas and of relatively early multisensory effects may accord with the increasing realization, throughout neuroscience, that sensory processing is not just a strictly serial progression through successive stages (Ungerleider and Mishkin, 1982; Rauschecker and Tian, 2000), but can involve many parallel and recursive loops (Kaas and Hackett, 2005; Scheich et al., 2007).

#### *Account C-F-C* (for ‘Critical Role of Feedback Circuitry’)

A further possible account, for at least some of the recently identified effects, is that multisensory influences on sensory-specific cortex may reflect *feedback* influences from multisensory convergence zones. This perspective could retain the traditional distinction between multisensory and sensory-specific regions, as defined by their feedforward inputs (e.g., Mesulam, 1998). But the former areas would now be able to influence even the latter, via feedback. Examples of such proposals arise, for instance, from Macaluso et al., 2000 (see also Macaluso et al., 2002; Macaluso and Driver, 2005), who found with fMRI that adding touch at the same location as a visual event boosted the BOLD response in human occipital visual cortex, within the contralateral lingual and fusiform gyrus. They attributed this to possible feedback influences from a tactile-visual convergence zone in parietal cortex, onto visual cortex, based on an analysis of effective connectivity (or “functional coupling”) for their fMRI data (see Figure 5C for schematic).

**Figure 4:**
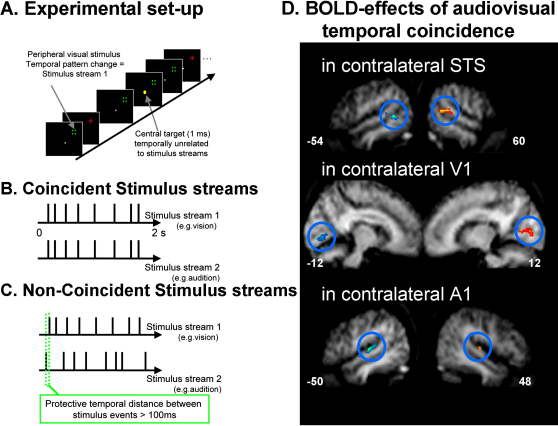
Multisensory Interactions in Humans Illustration of Noesselt et al. (2007) human fMRI study on audiovisual correspondence in temporal pattern (©2007 by the Society for Neuroscience, reprinted with permission). Schematics illustrating the stimulus set-up and design are shown at left, with illustrative group fMRI results on the right. (A) The top-left schematic illustrates a series of peripheral visual transients (change from green square to red cross, implemented inside the scanner with optic fibers) in the upper-right visual quadrant, while the participant fixates the lower central dot throughout, monitoring that for an occasional change in its brightness. During the stream of peripheral visual transients, a stream of auditory sound bursts (not shown in top schematic) could be emitted from a loudspeaker above the fixation point inside the scanner. (B and C) As shown in the two timeline schematics, visual and auditory streams each had erratic timing, and when both were present they either corresponded perfectly with each other (coincident temporal patterns, as in [B]) or had no temporal correspondence (as in [C]) despite comparable temporal statistics overall. (D) Relative to unimodal conditions (i.e., just visual or just auditory streams), audiovisual temporal correspondence (which is highly unlikely to arise by chance alone for these erratic temporal patterns) increased BOLD signal in superior temporal sulcus (STS, top brain image), contralateral to the corresponding visual stream (blue-green activation shown arises when that stream was in the right visual field, red-yellow activation when in the opposite visual field), whereas noncorrespondence decreased BOLD signal relative to the same unimodal baselines. Remarkably, an analogous pattern of results was also found for visual and auditory cortex (middle and bottom brain images), including primary areas (V1 and A1), even when considered at the level of each individual participant. Moreover, analyses of functional coupling and of directed information transfer between areas, for the BOLD data, indicated an influence from STS upon V1 and A1 that was significantly enhanced for the temporally corresponding condition, consistent with a possible feedback influence from STS.

**Figure 5:**
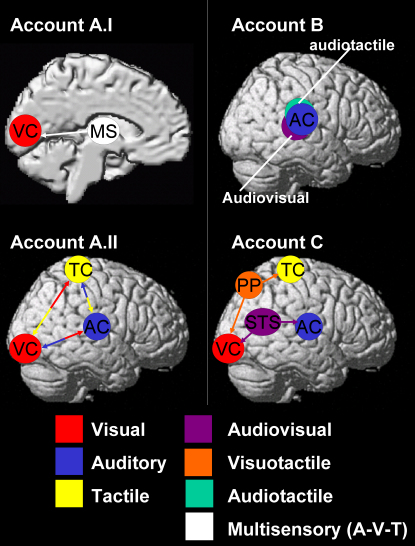
Possible Neural Pathways Mediating Multisensory Interplay, Shown Schematically to Make the Abstract Possibilities Discussed in the Main Text More Concrete (A) Direct feedforward influences between visual and auditory processing, which might either arise subcortically at thalamic levels, as sketched in (I), if multisensory (MS) thalamus influences visual cortex (VC); and/or via sparse cortical-cortical connections directly between auditory cortex (AC, blue), visual cortex (VC, red), and somatosensory or tactile cortex (TC, yellow), as in (II). (B) Some multisensory regions may exist near classic unisensory regions, as for some audio-visual areas (violet) and some audio-tactile (green) areas near conventional auditory cortex (blue). (C) Feedback connections may exist from higher-level multisensory regions, back to lower-level areas that are (predominantly) sensory specific apart from these feedback influences. For instance, visual and tactile modalities may interact via particular regions of posterior parietal cortex (PP, orange) that receive afferent input from both modalities and send feedback projections to each; and analogously, auditory and visual modalities may interact in posterior STS (violet) and send feedback projections to sensory-specific auditory and visual cortex. As discussed in the main text, while such potential architectures are often considered as rival views, in fact all of them may coexist. Future work needs to identify which particular pathways/architectures are causally involved in particular multisensory effects.

As a possible feedback example from the ERP domain, McDonald et al. (2003) studied modulation of visual ERPs by a task-irrelevant sound at the same or different location as a visual event. They suggested, based on source localization of ERP effects unfolding over time, that an initial crossmodal interaction arising in multisensory STS led to a subsequent effect in visual cortex, again apparently consistent with feedback influences (McDonald et al., 2005). Turning to invasive electrophysiological recordings in macaques, several studies have reported a relatively late modulation of A1 due to visual costimulation (Bizley et al., 2007; Brosch et al., 2005; Ghazanfar et al., 2005), with the latter authors speculating that this might involve feedback influences from STS. In a more recent human fMRI example, we observed (Noesselt et al., 2007) that audiovisual correspondence in temporal pattern may induce feedback influences from multisensory STS upon primary visual and auditory areas (see Figures 4 and 5C).

Finally, Bonath et al. (2007) used both fMRI and ERPs in humans to study the situation of spatial ventriloquism for sounds toward a co-occurring but displaced visual event (i.e., the very situation with which we began our review of perceptual/behavioral phenomena earlier). They reported that, for the same sound, BOLD signal in auditory cortex was smaller ispilateral to the visual location toward which that sound was mislocalized when ventriloquism arose (i.e., the left-right balance in auditory cortex was shifted in favor of the contralateral perceived location). ERPs also suggested modulation on those trials with ventriloquism, which shifted the left-right balance of ERPs attributed to a posterior auditory-cortex source, from around 200 ms poststimulus, again apparently consistent with a feedback influence.

We would not suggest that *all* multisensory phenomena arising in sensory-specific cortex will reflect feedback influences (see above for some direct evidence from invasive recordings for feedforward influences in certain cases). Nevertheless, a key testable prediction arises from specific “feedback” accounts, provided that these specify a particular source for the putative feedback influence, from an identified or candidate multisensory region, and/or via a particular route of intervening regions. If such models are correct, it should be possible to “knock out” the feedback effect on the sensory-specific regions affected, by lesioning or otherwise disrupting (e.g., with cooling, muscimol, or in humans using TMS) the hypothesized critical higher areas, while leaving the sensory-specific regions intact, to permit a measure of whether the multisensory influence is eliminated or is still present in those intact regions.

To our knowledge, as yet there are few extant examples of such approaches in the multisensory field, although examples do exist for testing feedback influences in this way for other hierarchical domains (e.g., between different levels of the visual system; see Bullier et al., 2001). Some notable lesion studies do exist on multisensory topics (e.g., Petrides and Iversen, 1978), but to our knowledge, these remain surprisingly sparse, despite the growing neuropsychological literature on possible multisensory effects in clinical patients (e.g., Bolognini et al., 2005; Farne et al., 2005; Ladavas et al., 2001). In one elegant multisensory animal study, Jiang et al. (2001) showed that cooling of multisensory cortices in cats (anterior ectosylvian cortex and pLS) eliminated the well-documented multisensory effects in SC neurons while leaving modality-specific discharges unaffected there. Such disruption of corticotectal influences also eliminated behavioral effects of multisensory stimulation for orienting responses (Jiang et al., 2002).

We anticipate that more multisensory studies will adopt such *causal* lesion/disruption/intervention approaches in the future, exploiting the increasing availability of methods for focal and reversible disruption, such as invasive microstimulation in animals (e.g., Graziano et al., 2002), cooling (e.g., Malhotra et al., 2004), and pharmacological manipulation (e.g., Smith et al., 2004) or transcranial magnetic stimulation (TMS) in humans (e.g., Ruff et al., 2006). A further key development for future invasive work will be to apply such manipulations to one particular region while recording the influence of this on remote but interconnected regions (see Ruff et al., 2006), as foreshadowed in the pioneering work of Jiang et al. (2001).

### Concluding Remarks

The field of multisensory research has rapidly expanded in recent years, with several new principles emerging in addition to many new opportunities for future research. First, numerous brain regions have been identified that receive input from multiple senses, both cortically and subcortically. In addition to well-known multisensory regions (as for SC, and as in STS, parietal, premotor and prefrontal cortex), these include some areas surprisingly close to primary sensory cortex, as shown in particular for regions in and around auditory cortex. Second, numerous fMRI and EEG/MEG studies have now shown that multisensory interplay can affect not only established multisensory convergence zones, but also brain areas and responses traditionally considered sensory specific. This accords with emerging psychophysical evidence that even sensory-specific judgments for one particular modality can sometimes be influenced by information entering a different sense. A variety of constraints on multisensory influences have been identified in both neural and psychophysical studies to date, including spatial, temporal, and more semantic/associative constraints, as reviewed more extensively elsewhere (e.g., Calvert et al., 2004; Macaluso and Driver, 2005; Ghazanfar and Schroeder, 2006; Spence and Driver, 2004).

A range of different accounts and architectures have been proposed for these newly uncovered phenomena, ranging from the rather extreme idea that all areas may be inherently multisensory (or perhaps less extremely, may all have at least some multisensory interneurons [Allman and Meredith, 2007] distributed among them, in differing proportions), to thalamic influences and/or direct connections between primary cortices, to the possibility that some multisensory effects may reflect feedback influences from higher-level multisensory convergence-zones, back to otherwise sensory-specific regions. Although such views have often been presented as rival alternatives, it is becoming increasingly clear that each may apply for a specific subset of phenomena, while critical methods for testing between the possibilities are now emerging. Nobody would dispute that sensory processing involves feedforward, lateral, and feedback connections. In the multisensory field, an important issue for the future is to identify the roles of these different types of circuit in specific multisensory phenomena and also to identify whether different types of neurons (e.g., predominantly unisensory cells, interdigitated with some multisensory interneurons) may be intermixed in some specific areas, potentially in different proportions that might then lead to a continuum, from predominantly sensory-specific to predominantly multisensory or even supramodal (see Dehner et al., 2004; Allman and Meredith, 2007).

A further intriguing issue for future work is that temporal, spatial, and semantic constraints on multisensory integration seem likely to arise at different points in time during sensory processing (in accord with the different time courses for extracting the relevant properties) and may therefore reflect distinct architectures. For instance, multisensory effects due merely to the presence/absence of costimulation in a second modality may arise more rapidly or automatically (and accordingly reflect a feedforward “sweep,” see Lakatos et al., 2007) than for multisensory effects due to more subtle relationships between information in the different senses or due to top-down factors involving feedback pathways. Likewise, the relative timing of inputs from different pairings of senses (e.g., auditory and visual or tactile and auditory) into particular brain regions will need to be considered (see Lakatos et al., 2007), and distinct circuits may underlie the interplay between distinct modality pairings (see Kayser et al., 2005, 2007; Ghazanfar and Schroeder, 2006).

The many new reports of multisensory influences on sensory-specific areas have also led to renewed interest in possible *plasticity* of sensory coding when a given sense is deprived, as for much recent work on brain responses for touch or sound in the blind (or blindfolded), or for vision and touch in the deaf, and so on (e.g., Bavelier et al., 2006; Bavelier and Neville, 2002; Merabet et al., 2004; Majewska and Sur, 2006; Roder et al., 1999). This is a rapidly expanding field that we cannot review in full here. We can note, however, that the literature on the normal brain that we have reviewed above already indicates that many regions that receive input primarily from one sense may also receive some direct or indirect inputs concerning other senses. Normally, these may function to modulate sensory-specific processing for the predominant modality in that area (e.g., boosting processing of a visual location in extrastriate cortex, when a visual event coincides with a sudden touch there, e.g., Macaluso et al., 2000; Macaluso and Driver, 2005). But when such areas become deprived of input from the usual predominant sense for them (as during blindness, or perhaps even during blindfolding), subtle or cryptic influences from other senses might then become further potentiated (e.g., as when visual cortex can come to respond to touch or audition in the congenital or early blind; Gougoux et al., 2005; Hunt et al., 2006; Sadato et al., 1996).

It is becoming increasingly clear that many multisensory phenomena may reflect causal interplay between remote but interconnected regions of the brain (Macaluso and Driver, 2005; Vuilleumier and Driver, 2007), rather than just the function of any single brain area(s). Timely new methodologies are now emerging for the study of such inter-regional interplay, including combination of local lesion, cooling, pharmacological modulation, or TMS applied to a given region, together with concurrent measures of functional neural activity remotely, in intact but interconnected regions, all studied in relation to ongoing behavior (e.g., Lomber and Galuske, 2002; Ruff et al., 2006). Future study of how the brain combines information from different senses is likely to require a correspondingly integrative combination of methods.
